# The Effects of Transcranial Direct Current Stimulation (tDCS) on Psychomotor and Visual Perception Functions Related to Driving Skills

**DOI:** 10.3389/fnbeh.2018.00016

**Published:** 2018-01-31

**Authors:** Alexander Brunnauer, Felix M. Segmiller, Sabine Löschner, Valérie Grun, Frank Padberg, Ulrich Palm

**Affiliations:** ^1^Department of Psychiatry and Psychotherapy, Klinikum der Universität München, Ludwig-Maximilians-University, Munich, Germany; ^2^Psychiatric Clinic, kbo-Inn-Salzach-Klinikum, Wasserburg am Inn, Germany

**Keywords:** tDCS, brain stimulation, dorsolateral prefrontal cortex, DLPFC, driving skills, driving performance

## Abstract

**Objective:** It could be demonstrated that anodal transcranial direct current stimulation (tDCS) of the left dorsolateral prefrontal cortex (DLPFC) enhances accuracy in working memory tasks and reaction time in healthy adults and thus may also have an influence on complex everyday tasks like driving a car. However, no studies have applied tDCS to psychomotor skills related to a standard driving test so far.

**Methods:** 10 female and 5 male healthy adults without any medication and history of psychiatric or neurological illness were randomly assigned to two groups receiving active and sham stimulation in a double blind, cross-over study design. Standardized computerized psychomotor tests according to the German guidelines for road and traffic safety were administered at baseline. Then they performed the same tests during an anodal or sham tDCS of the left DLPFC in two separated sessions.

**Results:** No significant improvements in skills related to driving performance like visual perception, stress tolerance, concentration, and vigilance could be shown after left anodal prefrontal tDCS. Side effects were low and did not differ between active and sham stimulation.

**Conclusions:** The findings of our study indicate that left prefrontal tDCS may not alter driving skills affording more automated action patterns but as shown in previous studies may have an influence on driving behavior requiring executive control processes. This however has to be proved in future studies and within greater samples.

## Introduction

Driving a car is considered an important part of daily life that embodies a complex and goal-directed task. This engages multiple interacting cognitive processes in different regions of our brain to maintain attention to traffic environment, focus on emerging information and threats, select and perform adequate reactions in terms of safety and traffic laws.

Most cognitive processes are modulated by the right and left dorsolateral prefrontal cortex (DLPFC), e.g., sustained attention (Pardo et al., 1991; Coull et al., 1998), error processing (Dosenbach et al., 2006), and planning (Unterrainer et al., 2005). However, left and right hemispheres seem to be responsible for different cognitive abilities. Thus, several neuroimaging studies addressed the specific functions of both hemispheres. The right DLPFC is involved in spatial tasks such as car-following and distance-keeping (Uchiyama et al., 2012), while the left DLPFC seems to be involved in tasks requiring sustained vigilance such as driving on a curved rural road with the need to pay attention to changing situations (Just et al., 2008).

In recent years, non-invasive brain stimulation methods such as transcranial direct current stimulation (tDCS) have been applied to focally change neuronal activation and its relevance in cognitive and behavioral performance. tDCS has been proven to change large-scale neuronal network function by application of weak direct current to the brain via a large electrode placed over the targeted brain regions (Keeser et al., 2011a,b) and has been shown to ameliorate symptoms in psychiatric disorders depending on its polarity (anodal and cathodal), e.g., depressive disorders (Palm et al., 2016). In neuropsychological studies, anodal and cathodal tDCS usually are applied to prefrontal and frontotemporal brain regions to assess the effects of inhibitory and excitatory stimulation on distinct neuropsychological functions and test performance. For example, in healthy adults it could be shown that anodal tDCS of the left DLPFC enhances accuracy in working memory tasks (Fregni et al., 2005; Zaehle et al., 2011), reaction time (Mulquiney et al., 2011; Teo et al., 2011), and declarative memory (Javadi and Walsh, 2012).

Only a few studies addressed the impact of tDCS on driving ability. Beeli et al. (2008) evaluated the impact of anodal and cathodal stimulation of the prefrontal cortex on risky driving behavior. They found that anodal stimulation of each right and left DLPFC but not cathodal stimulation resulted in a less risky driving style during a driving simulator test. The authors concluded that excitation of the right and left DLPFC caused stronger executive control and a more careful driving style. Sakai et al. (2014) found a better performance in car-following and lane-keeping after right-anodal/left-cathodal compared to left-anodal/right-cathodal tDCS. The authors conclude that this improvement is mediated by the enhancement of the right DLPFC where those spatial tasks are processed.

According to Michon (1989), driving behavior can be subdivided in three interacting hierarchical levels. A strategical level – e.g., choosing a route or consideration of road traffic rules, a tactical level – e.g., planning actions and maneuvre control, and an operational level with perceptual processing and action execution under high time pressure. Above mentioned studies (Beeli et al., 2008; Sakai et al., 2014) predominately investigated a more strategical and/or tactical level of driving behavior. The aim of the present study was to investigate the influence of left prefrontal tDCS on an operational level of driving behavior – i.e., visual perception, stress-tolerance, concentration, and vigilance. According to regulations of the German guidelines for road and traffic safety we focused on psychomotor- and visual perception-functions that are thought to be critical for an assessment of driving ability. The validity of these tests has been confirmed in large samples of both healthy controls and clinical samples. It could be demonstrated that a 83.3% correct classification for adjusted and unadjusted driving behaviors could be obtained with these tests (Bukasa et al., 1990; Karner and Biehl, 2001).

## Materials and Methods

### Subjects

This study was conducted at the Department of Psychiatry and Psychotherapy, Klinikum der Universität München, Germany, in accordance with the ethical standards laid down in the 1964 Declaration of Helsinki and its later amendments, and has been approved by the ethics committee of the Ludwig Maximilian University of Munich (No. 299-12). Written informed consent was obtained from all participants before enrolment. 15 healthy adults (10 female, 5 male) without any medication and any history of psychiatric and neurological illness were randomized to two groups receiving active and sham stimulation in a double blind, cross-over study design. Demographic variables and driving history were obtained from all subjects (see **Table 1**).

**Table 1:** Demographic variables and driving history, separated for early and late intervention group.

	Early	Late	Statistical
	intervention	intervention	significance^∗^
	(*n* = 8)	(*n* = 7)	
Age, mean (*SD*), y	33.1 (6.4)	31.0 (4.5)	NS
Gender, *n* (male/female)	3/5	2/5	NS
Civil status, *n*			
Unmarried	5	5	NS
Married	3	2	
Grammar or middle school	1	0	NS
High school or university diploma	7	7	
Driving license, mean (*SD*), y	14.5 (5.7)	14.9 (4.1)	NS
Yearly driven kilometers, mean (*SD*)	4500.0 (6546.5)	4428.6 (4961.7)	NS

*^∗^NS = not significant; y = years; SD = standard deviation.*

### Study Procedure

**FIGURE 1:**
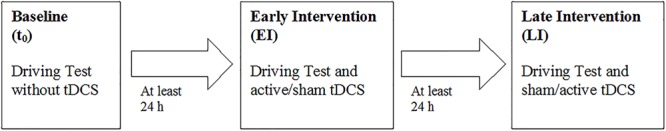
Study flow.

After given informed consent, a baseline assessment was carried out which included demographic variables and driving history. All subjects were tested in individual sessions at ∼9 am with standardized computerized psychomotor tests, according to the German guidelines for road and traffic safety. Complete testing lasted about 45–60 min depending on pace of work and was administered in the same sequence (visual perception – ATAVT, concentration – COG, stress-tolerance – DT, vigilance – VIGIL). To become familiar with the tests a training procedure (t_0_) was first conducted. To disentangle retest effects from stimulation effects, one group got active tDCS first, followed by sham (early intervention [EI]), the other in reversed order (late intervention [LI]). Psychomotor tests were performed parallel to the beginning of the stimulation (t_1_, t_2_). The training session and both interventional sessions were separated by an interval of at least 24 h to avoid carry-over effects (see **Figure 1**).

### Psychomotor and Visual Perception Tests

Several domains were assessed according to the German guidelines for road and traffic safety (Gräcmann and Albrecht, 2016), including visual perception, stress tolerance, concentration, and vigilance. According to these guidelines, a test has to be considered as failed if a participant falls short of the threshold of one standard deviation below the mean of normative data, derived from a representative sample of car drivers. The procedure has been described in detail elsewhere (Brunnauer et al., 2006). Data was collected using the computerized Wiener Testsystem (Vienna test system, WTS). It has been verified that more than 80% of subjects can be correctly classified according to adequate/inadequate driving behavior using results from this test system (Bukasa et al., 1990, 2003; Karner and Biehl, 2001).

*Visual perception* was assessed using the Tachistoscope Test (TAVT-MB; test-version S1). It measures the capability to perceive visual input quickly. Typical traffic situations are presented on 20 color slides for 1 s each followed by a multiple-choice question, containing five possible answers. The variable analyzed was the number of correct items; dependent on speed of operation the test lasted 10 min on average. The critical *Stress tolerance* was examined with the Wiener Determinationstest (Vienna determination test, DT; test-version S1). In three test phases the participant is presented with color, tone and light stimuli, 180 signals each. The interstimulus intervals vary within the three test phases. Subjects have to react by pressing corresponding buttons, bars and pedals using both their hands and feet; omissions in this test procedure were the critical variables; test procedure lasted 6 min. *Concentration* was measured with the attention and concentration test (COG; test-version S2). The task requires subjects to match simple figures with respect to similarity and dissimilarity. The test procedure lasted 8 min; the percentage of errors was the critical variable analyzed. The *Vigilance* Test (VIGIL; test version S1) requires the participant to remain attentive under monotonous conditions. A dot of light moving along a circle in fixed steps has to be observed over a period of 25 min. Irregularities – i.e., the dot skips over a circle – have to be identified by a keystroke. The variable analyzed was the number of correct items (i.e., number of stimuli minus omissions and errors).

### Transcranial Direct Current Stimulation

Transcranial direct current stimulation was applied with a CE-certified Eldith DC-Stimulator PLUS (NeuroConn, Ilmenau, Germany). This device delivers active or sham tDCS after entering a number code to achieve blinding of both operator and participant. The sham function mimics active stimulation by a short fade-in and fade-out phase (each 15 s) at the beginning and the end of the stimulation period (Palm et al., 2013). Current strength was set to 2 mA, duration of stimulation was 20 min with 15 s fade-in and fade-out. Saline-soaked sponge electrodes (35 cm^2^) were placed over the left DLPFC (anode, F3) and the contralateral supraorbital area (cathode, Fp2-Af8). Positioning of the electrodes was performed with a standard EEG cap according to the 10–20 international EEG system.

### Measurement of Side Effects

To control for potential side effects of the stimulation that could lead to unblinding of the participants, the Comfort Rating Questionnaire (CRQ) was used (Palm et al., 2014). This self-rating questionnaire assesses side effects (pain, tingling, burning, fatigue, nervousness, disturbed concentration, disturbed visual perception, headache) during and immediately after stimulation (sum scores) and general discomfort on a 10-point Likert scale ranging from “not at all” to “extremely.” Furthermore occurrence of visual flashes (phosphenes) and sleep disturbances after stimulation are assessed in a dichotomous question. As the test procedure outlasted the duration of stimulation, participants were advised to report their sensations in the first minutes and after the end of the test sequence.

### Data Analysis

Statistical analyses were performed using SPSS software (Statistical package for Social Sciences, Version 22, SPSS, Inc., Chicago, IL, United States). Demographic and clinical characteristics were analyzed using parametric and non-parametric tests (Chi-Square, Mann–Whitney *U*-test, *t*-tests). Due to different distributions and small sample size data from psychomotor assessments were z-transformed. A repeated measures analysis of variance was carried out separately for each functional domain (visual perception, concentration, stress tolerance, vigilance). Significant simple effects were localized with univariate *F*-tests. An alpha level of 0.05 was accepted as nominal level. To keep the type I error below this level, all *post hoc* tests were carried using the Sheffé test.

## Results

### Demographic Data

Demographic characteristics and driving history are provided in **Table 1**. There were no differences in gender, age, education, years of driving experience and driven kilometers between the EI and the LI group.

### Psychomotor and Visual Perception Tests

Multivariate analysis of variance was performed to assess effects of anodal prefrontal tDCS on functional domains relevant for driving behavior. With exception of the concentration test [*F*(1,13) = 6.4, *p* < 0.05] in the EI group, no significant alterations over time could be demonstrated in driving skills, nor in the EI group neither in the LI group. *Post hoc* Sheffé tests revealed, that significant time effects in the EI-group with respect to the concentration task could be seen in the sham and in the verum condition (all *p* < 0.05). Significant time-by-group effects, indicating specific stimulation effects were not found in both intervention groups (**Table 2**).

**Table 2:** Performance of participants in driving skills, separated for early and late intervention group.

Test	Results						Analysis					
	Early intervention (*n* = 8)			Late intervention (*n* = 7)			Early intervention (*n* = 8)			Late intervention (*n* = 7)		
							Time	Time × Group	Group	Time	Time × Group	Group
				
	t_0_	t_1_	t_2_	t_0_	t_1_	t_2_	*F* Statistic	*F* Statistic	*F* Statistic	*F* Statistic	*F* Statistic	*F* Statistic
**Visual Perception Test (TAVT)**												
Correct items (*SD*)	11.6 (3.0)	12.7 (3.1)	12.7 (2.8)	12.6 (3.8)	12.6 (3.7)	14.7 (3.8)	0.6	0.2	0.1	3.4	3.4	0.4
**Concentration Test (COG)**												
Percentage errors (*SD*)	2.5 (0.4)	2.3 (0.5)	2.4 (0.6)	2.4 (0.3)	2.1 (0.4)	2.1 (0.5)	6.4^∗^	0.5	0.5	0.1	0.1	0.8
**Stress Tolerance Test (DT)**												
Omissions (*SD*)	9.4 (3.3)	7.1 (2.8)	7.1 (3.1)	8.1 (3.2)	7.6 (4.1)	8.3 (5.0)	3.7	1.3	0.1	0.9	0.9	0.3
**Vigilance Test (VIGIL)**												
Correct items (*SD*)	96.1 (8.2)	97.9 (1.8)	96.6 (8.4)	99.7 (0.5)	98.8 (1.2)	98.6 (0.8)	0.1	0.8	1.6	0.3	0.1	0.6

*^∗^*p* < 0.05, SD = standard deviation.*

To sum up, no alterations on psychomotor skills relevant for driving could be demonstrated via anodal prefrontal tDCS stimulation in our sample.

### CRQ Results

During sham stimulation, mean sum score of side effects was 12.1 ± 3.4, after sham stimulation 9.0 ± 2.2. During active stimulation, mean sum score of side effects was 15.7 ± 9.5, after active stimulation 10.7 ± 3.9. Paired *t*-tests showed no significant difference in sum scores during active and sham and after active and sham stimulation (all *p* > 0.05).

Side effects were significantly lower after stimulation compared to during stimulation in the active (*p* < 0.05) and in the sham condition (*p* < 0.01). General discomfort showed no statistical significant difference between active (mean: 1.9) and sham (mean: 1.8) stimulation (*p* = n.s.). Sleep disturbances or phosphenes were not reported by any participant.

## Discussion

The aim of this study was to investigate the influence of left prefrontal tDCS on driving skills in a standardized driving test according to the German guidelines for road and traffic safety. 15 healthy adults without any medication and any history of psychiatric illness were randomized to two groups receiving active and sham stimulation in a double blind, cross-over study design.

This is – to our best knowledge – the first study investigating the influence of prefrontal tDCS on computerized psychomotor tests according to the German guidelines for traffic medicine and traffic psychology assessment. The main findings of our study are that no consistent improvements of driving skills like visual perception, stress tolerance, concentration, and vigilance could be found after active tDCS of the left DLPFC. tDCS was well-tolerated and there were low rates of discomfort after active and sham stimulation. It is therefore unlikely that side effects might have influenced the test performance.

According to Michon (1989), driving processes can be grouped into three interacting hierarchical levels: a strategical level and tactical level affording more executive control processes and an operational level with predominately automatic action patterns like action execution and perceptual processing. Albeit there is no conclusive model of neural substrates of driving behavior till now, there is however evidence, that these driving processes are associated with activations in specific brain regions (Spiers and Maguire, 2007). In contrast to other studies focusing on driving performance, we investigated the influence of left prefrontal tDCS on an operational level of driving behavior. Beeli et al. (2008) and Sakai et al. (2014) investigated a more strategic and tactical level of driving processes like risk behavior or longitudinal control. The study of Sakai reasoned that an upregulation of the right DLPFC (F4 anodal, F3 cathodal) improves vehicle control abilities in car-following and lane-keeping due to the processing of spatial tasks in the right hemisphere (Sakai et al., 2014). In a cross-over study with right and left anodal and cathodal tDCS, Beeli showed that anodal stimulation of both left and right DLPFC directly influences risky driving behavior when driving through a virtual environment in a driving simulator (anode F3 or F4, cathode on the ipsilateral mastoid) (Beeli et al., 2008). We investigated a more basic, operational level of driving processes requiring a high level of arousal to pass the test without failure. It is likely that because of high time pressure in these assessments more automatic response patterns are afforded compared to the studies of Beeli et al. (2008) and Sakai et al. (2014) where risky driving (speed, speed violation, distance, revolutions per minute) respectively fundamental vehicle control (car-following) were assessed, affording more anticipatory control processes. As Gill et al. (2015) outlined, the effects of a tDCS program on the DLPFC may be influenced by the cognitive demands of a task performed during stimulation.

There are some limitations to be considered in the interpretation of our study results. First of all, the number of investigated patients is, despite using cross-over design, rather small and a larger number could have brought different results. Second, healthy participants predominately were in an upper performance level and the speed tests used in our sample required a high level of arousal which probably could not have been increased by anodal stimulation because of ceiling effects. Moreover, prolonged stimulation at high intensity, particularly in a condition of activated cortex, can induce paradoxical homeostatic effects (e.g., Batsikadze et al., 2013), however the chosen parameters are sound to modulate prefrontal function. Third, the operational level explored includes psychomotor and cognitive functions, that are not under the strict competence of the cortical areas stimulated. Besides, electrode placement could have contributed to the negative results as the cathode was placed over right anterior-frontal regions and could have interfered in right DLPFC function. Another limiting factor could have been the duration of tests outlasting of about 25 min that of stimulation, although other studies reported positive results with neuropsychological test outlasting the stimulation for the same amount of time (e.g., Teo et al., 2011).

## Conclusion

Up to now, the relevance of different brain regions in driving performance and behavior remains unclear and the role of the interplay between both cortical hemispheres is not yet elucidated. Study designs and aims are heterogeneous and hamper comparability. Concerning tDCS studies on driving behavior, anodal tDCS of both hemispheres seems to improve risky driving whereas anodal tDCS of the right DLPFC seems to improve spatial functions. In our study we could not show an improvement in psychomotor and visual perception tests after anodal tDCS of the left DLPFC in psychomotor and visual perception functions related to driving skills, although improvement of cognitive functions by left-anodal tDCS has been shown in previous studies. Not least, with respect to rehabilitation efforts, there is a need for further studies on the effects of unilateral or bilateral tDCS on both hemispheres to elucidate the interplay of different neural substrates on specific processes of driving performance in clinical samples.

## Author Contributions

FS, AB, and UP designed the study and drafted the manuscript. VG performed tests and helped with manuscript preparation. SL and FP helped with scientific advice and proof reading.

## Conflict of Interest Statement

The authors declare that the research was conducted in the absence of any commercial or financial relationships that could be construed as a potential conflict of interest.
